# Relationship of Pain, Depression, Fatigue, and Sleep Problems with Functional Capacity, Balance, and Fear of Falling in Women with Fibromyalgia: Cross-Sectional Study

**DOI:** 10.3390/nursrep14040207

**Published:** 2024-10-08

**Authors:** Ángel Denche-Zamorano, Raquel Pastor-Cisneros, Pablo Tomas-Carus, José Carmelo Adsuar-Sala, Diana Salas-Gómez, Jose Alberto Parraca

**Affiliations:** 1 Promoting a Healthy Society Research Group (PHeSO), Faculty of Sport Sciences, University of Extremadura, 10003 Cáceres, Spain; denchezamorano@unex.es; 2 Departamento de Desporto e Saúde, Escola de Saúde e Desenvolvimento Humano, Universidade de Évora, 7004-516 Évora, Portugal; ptc@uevora.pt (P.T.-C.); jparraca@uevora.pt (J.A.P.); 3 Comprehensive Health Research Centre (CHRC), University of Evora, 7004-516 Évora, Portugal; 4 CIPER, Faculty of Human Kinetics, University of Lisbon, 1649-004 Lisbon, Portugal; 5 Movement Analysis Laboratory, Physiotherapy School Cantabria, Escuelas Universitarias Gimbernat (EUG), University of Cantabria, 39300 Torrelavega, Spain

**Keywords:** exercise, physical activity, physical fitness, rehabilitation, FMS

## Abstract

(1) Background: Fibromyalgia (FM) is a syndrome marked by chronic widespread pain, fatigue, sleep issues, and other symptoms. Interventions like physical exercise can potentially enhance physical function in individuals with FM. This study aimed to assess physical function, perceived physical fitness, balance confidence, and fear of falling in women with FM based on their levels of pain, depression, fatigue, and sleep problems. (2) Methods: Participants underwent a series of tests and questionnaires to evaluate physical and perceptual aspects. These included the Time Up and Go, Four Step Square Test, 6-Minute Walking Test, Maximum Handgrip Strength, Back Scratch, International Fitness Scale, Activities-Specific Balance Confidence Scale, and Fall Efficacy Scale—International. Participants were categorised by the severity of their pain, depression, fatigue, and sleep problems (mild, moderate, severe). A Kruskal–Wallis test assessed intergroup differences, while Spearman’s rho evaluated correlations between the study variables and symptom levels. (3) Results: Perceived physical condition varied significantly with symptom severity. Symptoms and sleep problems were notably linked to fear of falling, though no significant differences emerged in the physical tests. (4) Conclusions: In women with fibromyalgia, symptom severity was primarily related to perceptual and subjective aspects of physical condition and fall safety.

## 1. Introduction

Fibromyalgia (FM) is characterised by a range of functional symptoms, including chronic widespread pain, fatigue, sleep disturbance, and others [1,2]. As these symptoms combine to cause physical and psychological distress, people with FM have a reduced quality of life, reduced physical activity, reduced physical fitness, reduced physical function, or reduced ability to perform activities of daily living [3,4,5,6].

Despite affecting up to 5% of the world’s population [7], the aetiology of FM is still unknown [8,9]. It is known to be a complex syndrome with multiple contributing factors [10]. Among these, there is evidence of a genetic predisposition based on potential candidate genes that may be a risk factor for its occurrence [11,12]. Neurobiological factors have also been implicated in the development of the condition and its symptoms, such as alterations in neurotransmitters involved in pain control, central sensitisation, or hormonal changes [13,14,15]. In addition, the neurotransmitters that mediate pain transmission may also affect mood, memory, fatigue, and sleep [15].

Environmental factors such as chronic stress, lack of sleep, or personal experiences such as traumatic events also contribute to the development of FM [16]. Although men and women can experience similar symptoms [17], the prevalence between men and women is very unequal, and 80–96% of people with FM are women [7]. While some authors suggest that the prevalence of FM may be similar in men and women, men are less likely to be recognised and diagnosed [18,19].

Because of the many factors that contribute to the development and maintenance of fibromyalgia symptoms, a multimodal approach to treatment is required [11]. In this context, one of the non-pharmacological interventions supported by high-quality evidence for people with FM is exercise [9,11,20]. However, people with FM often have low levels of physical activity [21,22]. Fear of pain and kinesiophobia act as barriers to physical activity for people with FM, leading to an increased sedentary lifestyle, further deterioration in physical fitness, increased depression, and symptoms of FM, which feed off each other and contribute to the maintenance and progression of disability [23,24]. With low physical fitness, impaired physical function and low self-efficacy, there is increased mistrust or insecurity in one’s own abilities, such as lack of confidence in balance and self-perception of being able to perform physical activity [21,25], which in itself is a limiting factor for physical activity. For this reason, women with FM have a poorer self-perception of their physical condition than their subsequent performance in assessed physical tests, partly due to biopsychosocial factors such as catastrophic beliefs and lack of self-efficacy that these people have [21,26]. For example, in addition to balance problems, which can lead to an increased risk of falling, women with FM also have a fear of falling [24].

Symptoms such as widespread pain, depressive symptoms, fatigue, or sleep problems have been found to be associated with low levels of physical activity and reduced functional capacity in both the general population and people with FM [27,28,29]. It also appears that high or severe levels of pain are associated with low levels of physical activity and poorer performance on physical tests [30]. On the other hand, depressive symptoms are also associated with sedentary behaviour and loss of physical fitness [31]. Similarly, severe fatigue and sleep disturbances, such as unrefreshing sleep, have been shown to negatively affect people’s ability to be physically active and to impair their physical function [32]. In addition, sleep disorders can lead to daytime fatigue and affect mood and physical function [33].

On the other hand, in recent years, there has been a global health emergency that has posed an additional challenge for people living with a chronic condition. In particular, a recent study of Spanish women with fibromyalgia reported that during the pandemic, some women experienced an increase in the intensity and frequency of pain and some changes in their coping strategies. The COVID-19 pandemic had a significant impact on the quality of life of people with fibromyalgia and may have exacerbated their symptoms [34].

In view of the above, in order to be able to offer a specific approach to people with fibromyalgia in this new post-pandemic era through physical exercise, it is necessary to know how the relationship between the main symptoms of FM, such as the level of pain, depressive feelings, self-perceived fatigue, and sleep problems, are related to physical function, self-perceived physical condition, balance confidence and fear of falling in women with FM. Therefore, the main aim of this study was to assess physical function, perceived physical fitness, balance confidence, and fear of falling in women with FM according to the level of four of the main FM-related symptoms: pain, depression, fatigue, and sleep problems.

Thus, the initial hypotheses were the following:

Women with fibromyalgia who have higher levels of pain, greater depressive feelings, greater perceived fatigue, and greater self-reported sleep problems will have poorer physical function performance, lower perceived physical fitness scores, less confidence in their balance, and greater fear of falling.

In these people, there are inverse correlations between fibromyalgia symptoms and participants’ performance on physical tests, perceived physical fitness, and confidence in their balance, and direct correlations between fibromyalgia symptoms and fear of falling.

## 2. Materials and Methods

### 2.1. Design of the Study

This cross-sectional study followed the ethical guidelines of the Declaration of Helsinki and was approved by the Bioethics and Biosafety Committee of the University of Extremadura (approval number: 79/2018). Participants were informed about the nature of the study, the tests and questionnaires to be carried out, and the treatment of data. Before being included in the study, the women voluntarily signed informed consent forms. To ensure the identity of the women and the anonymity and confidentiality of the data, each participant was given an anonymous code to be kept during the study. The physical tests were performed in multi-purpose rooms at each of the four sites. Qualified technicians, graduates in physical activity and sport science, travelled to the sites to carry out the tests. Each participant was summoned to the appropriate site, and after receiving and signing the informed consent document, they were given an anonymous code and then performed the tests with the technicians. The questionnaires were completed by the participants in an online form before the physical tests were performed. The physical tests lasted approximately 1 h. These tests were carried out between January and April 2022. This study also complies with the STROBE recommendations for cross-sectional studies (Supplementary Materials: STROBE Statement—Checklist) [35].

### 2.2. Participants

The sample for this study consisted of Spanish adult women with FM. Local and national FM associations were contacted to recruit the sample, and participants were obtained from four sites in Spanish provinces: Cáceres, Elche, Madrid, and Palencia. To participate in the study, a number of inclusion criteria had to be met: being a woman, having been diagnosed with FM by a rheumatologist (meeting the criteria of the American Rheumatology Association [36]), not having any physical or cognitive limitations that prevented the performance of the physical tests or the completion of the study questionnaires, and not having any pathology or other condition that contraindicated the performance of physical exercise. Exclusion criteria were the following: taking psychotropic drugs or other drugs that could affect the results of the physical tests, suffering from neurodegenerative diseases, not signing the informed consent document, not being able to perform the physical tests or fill in the questionnaires. A total of 96 women were recruited, but 12 did not complete the questionnaires or take part in the physical tests. Therefore, the final sample consisted of 84 women with FM, aged between 20 and 72 years.

### 2.3. Variables and Instruments

Participants answered a socio-demographic questionnaire with questions about their Age, Civil Status, Employment situation, Smoking status, Drinking status, Years with FM symptoms, and Years since FM diagnosis.

#### 2.3.1. Subsequently, the Following Measurements or Tests Were Performed

Body Mass Index (BMI): In kg/m^2^. This was calculated after measuring the height and weight of the participants. The height of the participants without shoes was measured with a stadiometer (Seca 22, Hamburg, Germany). They were also weighed with a body composition analyser (TANITA MC 780 MA).

#### 2.3.2. Physical Tests

- *Time Up and Go (TUG):* The TUG is a basic functional mobility test in which participants are observed and timed to stand up from a chair (approximately 46 cm high), walk 3 metres, turn around a cone, walk backwards, and sit down again [37]. It is a simple and commonly used test to detect the risk of falling [38]. This test is valid and reliable in women with FM (ICC = 0.93) [39]. The same test was also performed in the imagined form (i-Time Up and Go. i-TUG), and with dual task (Dual Time Up and Go. Dual TUG). In the i-TUG, participants were asked to “Ready. In the i-TUG, participants had to visualise themselves performing the test and say “Go” as soon as they visualised themselves sitting back in the chair, the time was taken from the technician’s “Go” to the participant’s “Go” voice. In the Dual TUG test, participants had to perform the TUG while performing a mathematical task consisting of a two-by-two countdown from the number 50.
- *Four-Step Square Test (FSST):* The FSST [40], a valid and effective tool for measuring dynamic balance, standing mobility, and fall risk in older adults, was performed. This test has good to excellent reliability (ICC = 0.73–0.98) [41]. At the sound of “Ready. Now”, the participants had to perform a series of steps in different directions, following a given sequence [41]. The test was performed twice, timing both times and recording the seconds of the best test result.
- *4 m Walking Test (4mWT):* The 4mWT, a test that measures walking speed, with high reliability (ICC = 0.89–0.99), and which is used to estimate the participants’ risk of disability, was carried out [42,43]. At the sound of “Ready. Go”, the participant had to walk a distance of 4 metres at the fastest possible speed. The time taken by the participant to cover this distance was timed in seconds. The test was performed twice, and the best time was recorded.
- *30 m Walking Test (30mWT):* The 30 mWT, or Brisk Walking Test, was performed. This test measures walking speed [44]. The test has a high reliability (ICC = 0.93) and consists of walking 30 metres at the fastest possible speed. The test was performed twice and the best time in seconds was recorded.
- *6 min Walking Test (6mWT):* The 6 mWT was performed. This test consists of walking at the fastest possible sustained pace for 6 min around a rectangle with a perimeter of 45.7 metres [45]. The metres covered by the participants were recorded. This test is frequently used to determine the cardiorespiratory endurance [46]. It has been validated in women with FM (ICC = 0.92) [47].
- *30″ Sit to Stand Test:* This is a test used to assess lower-limb strength. The number of times participants managed to stand up and sit down from a chair for 30 s was recorded. Sitting in a chair with arms crossed over the chest, at the sound of “Ready. Now”, participants had to stand up and sit down as many times as they could during the test. This is a valid and reliable test in women with FM (ICC = 0.91). An imagined version (i-Sit to Stand) was also performed in which the participant had to visualise herself performing the test for 30 s, noting the number of repetitions imagined during this time. In addition, the test was performed with a dual task (Dual Sit to Stand), where the participant had to perform the test while counting down 2 by 2 from the number 100.
- *5 Sit to Stand repetitions:* This test consisted of timing in seconds how long the participants managed to get up and sit down from a chair. The participant started the test sitting in a chair with her arms crossed over her chest, and at the “Ready” signal, she had to stand up and sit down five times, as fast as she could. It is a test used to measure the functional strength of the lower limbs and has a good reliability (ICC = 0.81) [48].
- *Maximum Handgrip Strength:* A validated digital dynamometer (TKK 5101 Grip-D, Takey, Tokyo, Japan) [49] was used to measure the participants’ maximal handgrip strength. Standing with arms parallel to the trunk, participants were required to exert the greatest possible isometric hand force on the dynamometer. Two measurements were taken for each hand, with 1 min recovery time between each measurement. The highest force value between the four measurements was taken. This test was found to be valid and reliable (ICC = 0.95) [47]. The Strength/Weight Ratio was calculated by dividing the maximum force obtained in kg by the weight in kg of each participant.
- *30″ Biceps Curl Test:* This test was performed to assess the upper-limb strength of the participants, being a valid and reliable test in older adults (ICC = 0.92) [50]. The number of arm flexion–extensions that the participants were able to perform for 30 s, lifting a 2.3 kg weight, was recorded.
- *Back Scratch:* This was used to assess upper body flexibility. Two measurements were taken on each side (right and left), with the best measurement recorded in centimetres. The ability to reach over and under opposite shoulders and touch or overlap the fingers of opposite hands was measured. A negative number indicated the centimetres needed for the participant to overlap the hands [51], while positive numbers indicate the centimetres the participant managed to overlap the hands. This test is valid and reliable in women with FM (ICC = 0.96) [47].
- *Sit and Reach:* Lower-body flexibility was evaluated using the Chair Sit and Reach test [51]. Seated in a chair, the participant had to extend one leg and make a trunk flexion, trying to reach the tip of the foot with the tip of the toes. Two measurements were taken per side (right and left), and the best measurement was recorded in centimetres. Positive numbers indicate that the participant reached the tip of the foot with her toes, and negative values indicate the centimetres needed to reach the tip of the foot. This test is valid and reliable in women with FM (ICC = 0.94) [47].
- *Functional Reach:* This test measures the difference between the participant’s arm length and maximum forward reach, using a fixed support. It is used to assess balance. Two measurements were taken, and the best measurement was recorded in centimetres. This test has been validated and reliable (ICC = 0.92) [52].

#### 2.3.3. Questions and Questionnaires

- *Pain Level:* Participants were asked which alternative best described their usual level of pain and discomfort, with the following responses: 1: “Free of pain and discomfort”; 2: Mild or moderate pain that does not prevent activities; 3: “Moderate pain that prevents few activities”; 4: “Moderate or severe pain that prevents some activities”; 5: “Severe pain that prevents most activities”. For this research, responses were grouped into three levels of pain: Low (responses 1 and 2), Moderate (response 3), and Severe (responses 4 and 5).
- *Depression Feelings*: Participants were asked which statement best described their depressive feelings. Possible responses were as follows: 1: “I do not feel sad, melancholic or depressed at all”; 2: “I feel slightly sad, melancholic or depressed”; 3: “I feel moderately sad, melancholic or depressed”; 4: “I feel very sad, melancholic or depressed”; 5: “I feel extremely sad, melancholic or depressed”. For this research, responses were grouped into three levels of depressive feelings: Low (responses 1 and 2), Moderate (response 3), and Severe (responses 4 and 5).
- *Fatigue:* Participants were asked which statement best described their vitality. Possible responses were as follows: 1: “I feel healthy and energetic”; 2: “I feel slightly dejected, tired or weak”; 3: “I feel moderately dejected, tired or weak”; 4: “I feel very dejected, tired or weak”; 5: “I feel extremely tired, dejected or weak”. For this research, responses were grouped into three levels of fatigue: Low (responses 1 and 2), Moderate (response 3), and Severe (responses 4 and 5).
- *Sleep Problems:* Participants were asked which statement best described their ability to sleep. Possible responses were as follows: 1: “I am able to sleep normally, i.e., I have no problems with sleep”; 2: “I have mild problems with sleep, e.g., difficulty falling asleep, sometimes waking up at night”; 3: “I have moderate problems with sleep, e.g., distressing sleep or feeling that I have not had enough sleep”; 4: “I have major problems with sleep, e.g., I have the need to use sleeping pills often or routinely, or I wake up regularly at night and/or very early in the morning”; 5: “I suffer from severe insomnia, e.g., sleep is almost always impossible even using sleeping pills or I am awake most of the night”. For this research, responses were grouped into three levels of sleep problems: Low (responses 1 and 2), Moderate (response 3), and Severe (responses 4 and 5).
- *Fibromyalgia Impact Questionnaire (FIQ):* The FIQ [53] is a multidimensional questionnaire that assesses the impact of FM on the functional capacity and quality of life of people with FM, and the Spanish version was used in this study. The FIQ asks participants about their ability to perform daily living tasks in the last week, the number of days they felt well, days they were unable to work or do household chores because of FM, the difficulties they encountered in working, and the main symptoms of FM. It can take values between 0 and 100, where 0 indicates the highest functional capacity and quality of life, and 100 indicates the worst condition [54].
- *International Fitness Scale (IFIS):* IFIS [55] is a scale made up of five Likert-scale items that aim to know the self-perceived physical condition of the participants. The Spanish version of the IFIS was used and has been validated in women with FM [56]. The IFIS asks participants about their perception of their general physical condition, cardiorespiratory fitness, muscular strength, speed–agility, and flexibility compared to their age group, with a score between 1 and 5, where: 1: “Very poor”, 2: “Poor”, 3: “Average”, 4: “Good”, and 5: “Very good”. The total score of the IFIS was calculated by adding the scores of each of the items.
- *Activities-Specific Balance Confidence Scale (ABC Scale):* ABC Scale is an instrument that assesses people’s confidence in their balance [57]. Over the course of 16 items, participants were asked about their confidence in their balance in everyday life situations. The response options range from 0% confidence to 100% confidence. The overall score of the ABC Scale is the mean percentage of the items answered and can take values from 0 to 100, with 0 being the worst confidence in balance and 100 being the best. The participants responded to the Spanish version of this instrument, a valid and reliable version [58].
- *Fall Efficacy Scale—International (FES—I):* FES—I is a scale that assesses the participants’ level of concern about falling when performing tasks of daily living. It consists of 16 items with a response range between 1 and 4, where 1 indicates not at all concerned, and 4 indicates very concerned. It can take values between 16 and 64, where 16 indicates not at all worried, and 64 indicates very worried. FES—I is a valid and reliable instrument (ICC = 0.96) [59,60,61]. In this study, the validated Spanish version was used [61].

### 2.4. Statistical Analysis

Normality was studied with a Kolmogorov–Smirnov test. A descriptive analysis was performed, presenting the sample in terms of median and interquartile range (IQR) (continuous variables: Age, BMI, Years since diagnosis, Years with symptoms and Fibromyalgia impact), and absolute and relative frequencies (categorical variables: pain level, depression, fatigue, and sleep problems). The median and IQR of the results of physical tests and physical fitness questionnaires (IFIS and its components: General Fitness, Cardiorespiratory Fitness, muscular strength, speed–agility, and Flexibility), confidence in their balance (ABC Scale), and fear of falling (FES—I) were calculated and presented as a function of: Pain levels, Depression levels, Fatigue levels, and Sleep Problem levels. The Kruskal–Wallis test was used to test for significant intergroup differences. Spearman’s rho was calculated to assess the correlations between the study variables and the levels of the four fibromyalgia symptoms. In all analyses, a significance level of *p* < 0.05 was established with the Bonferroni correction setting the significance level at 0.002. Analyses were performed with the statistical software IBM SPSS Statistics for Windows, Version 25.0. Armonk, NY: IBM Corp. and Microsoft Excel version 2405.

## 3. Results

The main characteristics of the sample are presented in Table 1. It can be seen that the sample had a median age of 57 years (IQR = 12), reported having symptoms for 20 years (IQR = 20), a median number of years since diagnosis of the disease of 12 years (IQR = 14), and an impact of fibromyalgia of 62.4 (IQR = 22.3).

Table 1 shows the distribution of participants according to fibromyalgia symptoms: degree of pain, depression, fatigue, and sleep problems. In total, 69.0% and 10.7% of the sample presented a moderate to severe level of pain, affecting four out of five participants; 45.8% had moderate depressive feelings, with 8.4% having severe depressive feelings. In terms of fatigue, 64.3% were moderately fatigued, with 14.3% being severely fatigued. Finally, 64.3% of the sample had moderate sleep problems, with 10.7% having severe problems.

Moreover, a total of 89.3% of the participants were nonsmokers, compared to only 10.7% who were smokers. A total of 67.9% stated that they never or rarely drank alcohol. In contrast, 17.9% reported drinking alcohol occasionally, and 14.3% drank alcohol frequently or very frequently. Finally, 42.9% were working women, the remaining participants reported being retired (31.0%), unemployed (23.8%), or a housewife (2.4%).

### 3.1. Functional Capacity in Women with Fibromyalgia as a Function of Pain Level

Table 2 shows the relationship between functional test results and pain level in women with fibromyalgia. A higher test performance was found in those with a lower pain level than in those with higher pain levels, except for the functional reach test. We also found a higher perception of general physical fitness and all its components in people with lower levels of pain, as well as greater confidence in balance and less fear of falling. However, significant differences were only found in the TUG, FSST, IFIS, General Fitness, speed–agility, and FES—I. Weak to moderate correlations were found between pain level and all other variables. In addition, only direct significant correlations were found between pain level and performance in the TUG (rho: 0.381), FSST (rho: 0.452), and 5rep Sit to Stand Test (rho: 0.392), in all of which the time taken to complete the tests increased as the degree of pain presented by the participants increased (*p* < 0.001).

Significant inverse correlations were also found between pain level and IFIS (rho: −0.455), General Fitness (rho: −0.472), speed–agility (rho: −0.524), and ABC Scale (rho: −0.318) scores, all with *p* < 0.001. Perceived physical fitness and balance confidence were worse as participants’ pain level increased. In contrast, a direct relationship was found between pain level and FES—I scores (rho: 0.474, *p* < 0.001), with fear of falling being higher with increasing pain.

### 3.2. Functional Capacity in Women with Fibromyalgia as a Function of Depressive Feelings

Table 3 shows the relationship between functional test scores and depressive feelings. Women with low depressive feelings performed better on all physical tests than those with higher depressive feelings. However, significant differences were only found between depressive feelings and self-perceived physical fitness: IFIS (*p* < 0.001), General Fitness (*p* < 0.001), and muscular strength (*p* < 0.001). In addition, significant differences were also found in confidence in balance (*p* < 0.001) and fear of falling (*p* < 0.001). Perceived physical fitness was higher in people with lower feelings of depression, as well as lower fear of falling. Conversely, people with greater feelings of depression had less confidence in their balance. Weak to moderate correlations were found between feelings of depression and: IFIS (rho: −0.495, *p* < 0.001), General Fitness (rho: −0.432, *p* < 0.001), muscular strength (rho: −0.432, *p* < 0.001), speed–agility (rho: -.393, *p* < 0.001), and FES—I (rho: 0.535, *p* < 0.001).

### 3.3. Functional Capacity in Women with Fibromyalgia as a Function of Perceived Fatigue

Table 4 shows the participants’ performance in the physical tests, perceived physical fitness, confidence in their balance, and fear of falling as a function of perceived fatigue. In all physical tests, better results were found for those with low perceived fatigue. However, no significant differences were found. Significant differences were found between perceived fatigue and perceived physical fitness and some of its components: IFIS (*p* < 0.001), General Fitness (*p* < 0.001), Cardiorespiratory Fitness (*p* < 0.001), muscular strength (*p* < 0.001), and speed–agility (*p* < 0.001). In addition, significant differences were found between FES—I scores (*p* < 0.001). Moderate correlations were found between perceived fatigue and perceived physical fitness: IFIS (rho: −0.562, *p* < 0.001), General Fitness (rho: −0.572, *p* < 0.001), Cardiorespiratory Fitness (rho: −0.413, *p* < 0.001), muscular strength (rho: −0.483, *p* < 0.001), and speed–agility (rho: −0.429, *p* < 0.001). In addition, a moderate direct correlation was found between perceived fatigue and fear of falling (rho: 0.570, *p* < 0.001).

### 3.4. Functional Capacity in Women with Fibromyalgia as a Function of Sleep Problems

Finally, Table 5 showed the participants’ performance in the physical tests, perceived physical fitness, confidence in their balance, and fear of falling as a function of self-reported sleep problems. Despite finding better performance in all physical tests in people with low self-reported sleep problems, no significant inter-group differences were found. Only significant differences were found between sleep problems and fear of falling (*p* < 0.001). Furthermore, a moderate direct correlation was found between these two variables (rho: 0.471, *p* < 0.001).

## 4. Discussion

This study aimed to assess physical function, perceived physical fitness, balance confidence, and fear of falling in women with fibromyalgia as a function of degree of pain, depressive feelings, fatigue, and sleep problems in women with fibromyalgia.

The main finding of this study was that statistically significant differences were found in perceived physical fitness, especially in general physical fitness as a function of degree of pain, depressive feelings, and perceived fatigue. In addition, statistically significant differences were also found in fear of falling as a function of these same symptoms and sleep problems.

People with a higher degree of pain had a worse perception of their physical condition, especially their general condition and their speed–agility. This perception on the part of the participants was supported by the results of the physical tests. People with a higher degree of pain performed worse in the physical tests. Similar results were obtained by Dailey et al. in previous research, in which pain was shown to be associated with perceived physical fitness as well as physical performance in women with fibromyalgia [62]. These findings were also supported by a previous study that reported an association between higher physical fitness and lower pain in this population [63]. Significant intergroup differences were found between TUG, ranging from 6.39 s in people with low pain to 10.12 s in people with high pain. This deterioration in gait speed as a function of pain grade in women with fibromyalgia was also reported by another study [64]. Differences in FFST were also found, from 5.45 s in people with low pain to 10.08 s in people with high pain. It has been shown that the FFST can be a good instrument for the prevention of falls in this population [65], and taking into account that the degree of pain is related to a greater tendency to fall [66], one can find the justification that those women with less pain obtained a better result, which translates into a shorter time in the FFST. This tendency was found in all physical tests. Only in the functional reach, no better results were found in those with lower pain than in those with higher pain. In contrast to our results, other studies did associate a better functional reach test score or a higher level of flexibility with a lower degree of pain [67,68], probably because the sample of participants in these studies was considerably larger. Despite all these trends, no significant differences or significant correlations were found for many of the variables assessed.

Similar findings were found for depression. People with severe depressive feelings had worse-perceived physical fitness, greater fear of falling, and also less confidence in their balance. With regard to perceived physical fitness, significant associations were found between a higher stage of depressive severity and worse-perceived physical fitness [69], as well as research finding the same relationship between both variables in women with fibromyalgia [70,71]. Furthermore, it has been shown that the rate of falls is associated with depressive symptoms [72], as well as the rate of falls is related to the fear of falling in this population [24], which is in line with our study that associates women with fibromyalgia and greater feelings of depression with a greater fear of falling. Considering the relationship between fear of falling and balance confidence in this population [73], our findings are reaffirmed by the research of Dylan et al. who found an association between depressive symptoms and objective postural control performance [74]. The study by Loren et al. indicated that healthy balance is associated with better psychological profiles in fibromyalgia [75], reinforcing our findings.

Regarding fatigue, our study found significant differences between perceived fatigue and some components of perceived physical fitness. In this regard, a previous study by Alvarez et al. found relationships between higher fatigue symptom scores and worse self-reported physical activity in fibromyalgia patients [76]. It has been shown that poorer self-reported physical activity is significantly associated with poorer perceived physical fitness [77], thus affirming the evidence found by the present study. Specifically in women, the influence of perceived fatigue on the decline of muscular strength has been demonstrated [78]. At the same time, positively perceived fatigue implies higher levels of general fitness as well as agility in women with fibromyalgia [79]. In addition, higher perceived fatigue is associated with a higher rate of falls [79], as well as lower walking confidence [80], which may translate into a greater fear of falling. Notably, in women with fibromyalgia, perceived fatigue is considered a potential risk factor for falls [81].

In the present study, self-reported sleep problems were significantly associated with fear of falling. In Spanish women, there is evidence of a relationship between poor sleep quality and/or lack of sleep with poor postural stability [82]. In addition, a previous study reported a significant association between poor sleep health and an increased fear of falling and, consequently, an increased risk of falling [83]. In women with fibromyalgia, a close relationship between sleep quality with functional status and frequency of falls has been reported [84], as well as with fear of falling [23], i.e., sleep problems are associated with a worsening of functional status which consequently increases the frequency of falls and fear of falling, in line with the results reported in our study.

### 4.1. Limitations

The main limitation lies in the design of the study itself, due to the fact that the temporal cross-sectional nature of the study does not allow for a cause–effect relationship to be established between the variables, and inverse causality cannot be ruled out. Another limitation could be related to the large number of tests carried out and in turn to the limited sample analysed, which may have conditioned the results and limited the statistical capacity of the study to detect significant differences or correlations between the variables analysed. These limitations are the starting point for future studies with a larger representative sample of the population with fibromyalgia that will allow us to detect the most sensitive tests for detecting the impact of fibromyalgia symptoms on functional capacity. Taking into account the various symptoms associated with fibromyalgia, such as high self-reported fatigue, which has also been highlighted in this study, these future studies will serve to refine and optimise measurement protocols in this population. Furthermore, the development of longitudinal studies with a large representative sample will allow us to extrapolate the results to the community of women with fibromyalgia, as well as to establish the temporal sequence between the variables assessed in this study and to observe the real impact of the symptoms of people with fibromyalgia on their functional capacity. In future research, especially with larger samples, it would be necessary to include statistical models that incorporate possible confounding variables such as age, other pathologies, or severity of pathologies. These variables could have influenced the results of our investigation and is another limitation of our study.

### 4.2. Practical Implications

It has been shown that the prevalence of sleep and balance disorders is higher in women with fibromyalgia [84], so, taking into account that these factors are related to the fear of falling and the risk of falling, and taking the results of this study as a reference point, programmes that favour the control and monitoring of these variables in this population should be promoted. Therefore, this study provides information that may be useful for health professionals: doctors, nurses, etc., who, from a health perspective, address these symptoms (pain, depression, fatigue, and sleep problems) in women with fibromyalgia, knowing the relationship they have with their functionality, confidence in their balance, and fear of falling. These symptoms are also related to their ability to perform daily living tasks or household chores.

At the same time, this study also shows a worsening of perceived physical condition according to the degree of disease symptomatology: pain, depressive feelings, and perceived fatigue. This fact demonstrates the need to include physical activity as a non-pharmacological intervention.

Therefore, the results can be used to promote the health, mental health, and well-being of women with fibromyalgia in order to improve their quality of life.

## 5. Conclusions

Statistically significant differences were found in perceived physical condition as a function of degree of pain, depressive feelings, and perceived fatigue in women with FM. Significant associations were also found between these symptoms and sleep problems with respect to fear of falling in this population. Pain was found to be strongly associated with objective physical tests (TUG, FSST, 5rep sit to stand, and hand grip), and a higher degree of pain was associated with worse performance on these tests. Also, participants with severe depressive feelings had poorer perceived physical condition, greater fear of falling, and also less confidence in their balance. No significant evidence was found between the physical tests assessed as a function of these symptoms, so a dependency relationship between these symptoms and worse physical function performance in patients with FM could not be established. These findings may be due to a limited sample and the large number of tests performed. Based on our results and the sample analysed, it seems that the level of FM symptoms is mostly associated with the perceptual and subjective aspects related to physical fitness and fall safety of women with FM. Further research and investigation in this field would be necessary to know the real impact of fibromyalgia symptoms on the physical function of women with FM.

## Figures and Tables

**Table 1:** Characterisation of the sample.

Variables	Total (*n* = 84)	
	Median	(IQR)
Age (Years)	57.0	(12.0)
BMI (kg/m^2^)	28.6	(10.3)
Years since diagnosis	12.0	(14.0)
Years with symptoms	20.0	(20.0)
FIQ (Score 0–100)	62.4	(22.3)
Pain Level	*n*	%
Low	17	25.0
Moderate	58	69.0
Severe	9	10.7
Depression		
Low	38	45.8
Moderate	38	45.8
Severe	7	8.4
Fatigue		
Low	18	21.4
Moderate	54	64.3
Severe	12	14.3
Sleep Problems		
Low	21	25.0
Moderate	54	64.3
Severe	9	10.7

*n* (Participants); IQR (Interquartile range); BMI (Body Mass Index); IFIS (International Fitness Scale. 0: the worst self-reported fitness; 25: the best self-reported fitness); FIQ (Fibromyalgia Impact Questionnaire. From 0 to 100 indicating the lowest to highest impact); % (Percentage).

**Table 2:** Relationships and correlations between level of pain, physical function, perceived physical fitness, balance confidence, and fear of falling.

Variables	Pain Level							Correlations	
	Low (*n* = 17)		Moderate (*n* = 58)		Severe (*n* = 9)				
	Mdn	(IQR)	Mdn	(IQR)	Mdn	(IQR)	*p*	*rho*	*p* †
i-Time Up and Go test (s)	5.06	(1.96)	5.34	(2.64)	7.10	(3.97)	0.363	0.155	0.159
Time Up and Go test (s)	6.39	(0.63)	7.47	(3.20)	10.12	(11.68)	0.001 *	0.381	<0.001 ***
Dual Time Up and Go test (s)	7.78	(2.06)	9.03	(4.91)	10.71	(7.5)	0.058	0.251	0.021
Four Step Square test (s)	5.45	(1.48)	7.44	(2.03)	10.08	(3.63)	<0.001 ***	0.452	<0.001 ***
4 m Walking test (s)	2.47	(0.71)	2.66	(0.65)	3.34	(0.75)	0.007	0.295	0.007
30 m Walking test (s)	17.35	(3.76)	19.15	(6.56)	23.24	(10.59)	0.125	0.196	0.081
6 min Walking test (m)	471	(101)	483	(159)	383	(141)	0.265	−0.124	0.271
i-30″ Sit to Stand test (rep)	17	(7)	12	(5)	12	(5)	0.022	−0.236	0.031
30″ Sit to Stand test (rep)	12	(2)	10	(4)	8	(6)	0.012	−326	0.003
Dual 30″ Sit to Stand test (rep)	10	(2)	9	(4)	8	(5)	0.043	−0.275	0.011
5rep Sit to Stand test (s)	11.88	(3.00)	13.20	(4.00)	19.87	(12.00)	0.002	0.392	<0.001 ***
Max Strength Handgrip (kg)	23.22	(9.45)	19.90	(20.80)	17.60	(13.85)	0.004	−0.363	0.001 *
Max Strength/Weight Ratio	0.39	(0.15)	0.28	(0.11)	0.32	(0.21)	0.026	−0.294	0.023
30″ Biceps Curl test (rep)	17	(6)	13	(7)	15	(8)	0.069	−0.219	0.048
Functional reach test (cm)	32	(10)	35	(13)	36	(14)	0.517	0.116	0.293
Chair Sit and Reach (cm)	2	(21)	−2	(10)	−10	(20)	0.117	−0.227	0.042
Back Scratch (cm)	−3	(11)	−4	(14)	−13	(14)	0.207	−0.147	0.183
IFIS (Score 5–25)	14.0	(4.0)	12.0	(3.0)	9.0	(5.0)	<0.001 ***	−0.455	<0.001 ***
IFIS. General Fitness (Score 1–5)	3.0	(1.0)	2.0	(1.0)	2.0	(0.5)	<0.001 ***	−0.472	<0.001 ***
IFIS. Cardiorespiratory Fitness (Score 1–5)	3.0	(2.0)	3.0	(1.0)	3.0	(1.5)	0.067	−0.249	0.022
IFIS. Muscular Strength (Score 1–5)	3.0	(2.0)	2.0	(1.0)	2.0	(1.5)	0.053	−0.247	0.024
IFIS. Speed–Agility (Score 1–5)	3.0	(1.0)	2.0	(1.0)	2.0	(0.0)	<0.001 ***	−0.524	<0.001 ***
IFIS. Flexibility (Score 1–5)	3.0	(1.0)	2.5	(1.0)	2.0	(2.0)	0.097	−0.229	0.036
ABC Scale (Score 0–100)	63	(28)	53	(28)	42	(34)	0.015	−0.318	<0.001 ***
FES—I (Score 16–64)	27	(10)	39	(16)	44	(11)	<0.001 ***	0.474	<0.001 ***

*n* (participants); Mdn (Median); IQR (Interquartile Range); *p* (*p*-value from Mann–Whitney U test, Bonferroni correction *p* < 0.002); *p* † (*p*-value from Spearman’s rho, Bonferroni correction *p* < 0.002); * (*p* < 0.002); *** (*p* < 0.001); s (Seconds); Rep (Repetitions); m (Metres); cm (centimetres); i (Imagined); Max (Maximum); IFIS (International Fitness Scale. 5: the worst self-reported fitness; 25: the best self-reported fitness. IFIS Components: Score 1–5, with 1 being the worst self-reported fitness and 5 being the best); ABC (Activities-Specific Balance Confidence Scale. 0: Not confident at all; 100: completely confident); FES—I (Fall Efficacy Scale—International. 16, no concern about falling; 64, severe concern about falling).

**Table 3:** Relationships and correlations between depressed feelings, physical function, perceived physical fitness, balance confidence, and fear of falling.

Variables	Depressed Feelings							Correlations	
	Low (*n* = 38)		Moderate (*n* = 38)		Severe (*n* = 7)				
	Mdn	(IQR)	Mdn	(IQR)	Mdn	(IQR)	*p*	*rho*	*p* †
i-Time Up and Go test (s)	5.21	(2.45)	5.35	(3.42)	5.59	(4.32)	0.475	0.131	0.238
Time Up and Go test (s)	6.61	(1.72)	8.69	(4.05)	10.06	(14.55)	0.015	0.314	0.004
Dual Time Up and Go test (s)	8.16	(3.05)	9.97	(6.45)	11.54	(21.49)	0.025	0.298	0.006
Four Step Square test (s)	6.68	(2.36)	7.33	(2.47)	8.45	(7.65)	0.048	0.262	0.017
4 m Walking test (s)	2.47	(0.57)	2.85	(0.87)	3.53	(3.19)	0.043	0.280	0.011
30 m Walking test (s)	18.50	(4.65)	19.65	(6.22)	23.91	(16.96)	0.346	0.161	0.156
6 min Walking test (m)	483	(118)	452	(164)	397	(301)	0.234	−0.192	0.090
i-30″ Sit to Stand test (rep)	14	(8)	12	(5)	13	(12)	0.076	−0.237	0.031
30″ Sit to Stand test (rep)	12	(3)	9	(3)	9	(13)	0.007	−0.333	0.002
Dual 30″ Sit to Stand test (rep)	10	(2)	8	(4)	7	(10)	0.022	−0.297	0.006
5rep Sit to Stand test (s)	12.36	(2.00)	14.14	(7.00)	11.97	(11.00)	0.011	0.245	0.026
Max Strength Handgrip (kg)	23.41	(5.60)	21.45	(6.98)	23.30	(8.60)	0.097	−0.197	0.074
Max Strength/Weight Ratio	0.36	(0.15)	0.30	(0.12)	0.26	(0.15)	0.056	−0.314	0.015
30″ Biceps Curl test (rep)	15	(7)	13	(7)	15	(18)	0.238	−0.139	0.216
Functional reach test (cm)	34	(12)	35	(13)	32	(14)	0.725	0.009	0.938
Chair Sit and Reach (cm)	0	(14)	−2	(13)	−4	(9)	0.456	−0.134	0.235
Back Scratch (cm)	−2	(11)	−10	(15)	−6	(16)	0.087	−0.238	0.030
IFIS (Score 5–25)	14.0	(3.0)	11.0	(3.0)	11.0	(5.0)	<0.001 ***	−0.495	<0.001 ***
IFIS. General Fitness (Score 1–5)	3.0	(1.0)	2.0	(1.0)	2.0	(0.0)	<0.001 ***	−0.432	<0.001 ***
IFIS. Cardiorespiratory Fitness (Score 1–5)	3.0	(1.5)	3.0	(1.0)	3.0	(1.0)	0.069	−0.250	0.022
IFIS. Muscular Strength (Score 1–5)	3.0	(1.0)	2.0	(0.0)	2.0	(2.0)	<0.001 ***	−0.432	<0.001 ***
IFIS. Speed–Agility (Score 1–5)	3.0	(1.0)	2.0	(1.0)	2.0	(1.0)	0.002	−0.393	<0.001 ***
IFIS. Flexibility (Score 1–5)	3.0	(1.0)	2.0	(1.0)	3.0	(2.0)	0.076	−0.236	0.032
ABC Scale (Score 0–100)	58	(34)	54	(20)	39	(54)	<0.001 ***	−0.269	0.014
FES—I (Score 16–64)	30	(11)	42	(12)	46	(9)	<0.001 ***	0.535	<0.001 ***

*n* (participants); Mdn (Median); IQR (Interquartile Range); *p* (*p*-value from Mann–Whitney U test, Bonferroni correction *p* < 0.002); *p* † (*p*-value from Spearman’s rho, Bonferroni correction *p* < 0.002); *** (*p* < 0.001); s (Seconds); Rep (Repetitions); m (metres); cm (centimetres); i (Imagined); Max (Maximum); IFIS (International Fitness Scale. 5: the worst self-reported fitness; 25: the best self-reported fitness. IFIS Components: Score 1–5, with 1 being the worst self-reported fitness and 5 being the best); ABC Scale (Activities-Specific Balance Confidence Scale. 0: Not confident at all; 100: completely confident); FES—I (Fall Efficacy Scale—International; 16, no concern about falling; 64, severe concern about falling).

**Table 4:** Relationships and correlations between fatigue, physical function, perceived physical fitness, balance confidence, and fear of falling.

Variables	Fatigue							Correlations	
	Low (*n* = 18)		Moderate (*n* = 54)		Severe (*n* = 12)				
	Mdn	(IQR)	Mdn	(IQR)	Mdn	(IQR)	*p*	*rho*	*p* †
i-Time Up and Go test (s)	4.47	(3.38)	5.34	(2.39)	5.82	(4.33)	0.261	0.162	0.141
Time Up and Go test (s)	6.72	(1.67)	7.45	(3.26)	9.08	(5.75)	0.214	0.193	0.079
Dual Time Up and Go test (s)	8.05	(1.90)	9.06	(4.73)	11.83	(12.01)	0.085	0.243	0.026
Four Step Square test (s)	5.91	(2.41)	7.32	(2.01)	7.74	(4.93)	0.055	0.230	0.036
4 m Walking test (s)	2.66	(0.56)	2.64	(0.81)	3.06	(1.26)	0.394	0.142	0.202
30 m Walking test (s)	19.82	(5.88)	17.90	(5.18)	23.91	(15.43)	0.207	0.050	0.662
6 min Walking test (m)	454	(102)	483	(143)	397	(214)	0.254	0.024	0.835
i-30″ Sit to Stand test (rep)	14	(8)	12	(5)	12	(12)	0.239	−0.178	0.106
30″ Sit to Stand test (rep)	12	(3)	10	(3)	9	(10)	0.032	−0.267	0.014
Dual 30″ Sit to Stand test (rep)	10	(3)	10	(3)	8	(9)	0.171	−0.206	0.060
5rep Sit to Stand test (s)	12.09	(2.00)	13.56	(4.00)	14.62	(13.00)	0.145	0.153	0.166
Max Strength Handgrip (kg)	24.40	(6.55)	22.10	(5.30)	21.35	(12.50)	0.032	−0.276	0.011
Max Strength/Weight Ratio	0.36	(0.14)	0.30	(0.14)	0.26	(0.19)	0.045	−0.321	0.013
30″ Biceps Curl test (rep)	16	(6)	14	(6)	13	(9)	0.245	−0.173	0.120
Functional reach test (cm)	36	(9)	34	(13)	31	(10)	0.641	−0.063	0.569
Chair Sit and Reach (cm)	2	(9)	−2	(13)	−4	(21)	0.064	−0.254	0.022
Back Scratch (cm)	−2	(15)	−5	(13)	−12	(20)	0.127	−0.211	0.054
IFIS (Score 5–25)	15.5	(4.0)	12.0	(4.0)	11.0	(4.0)	<0.001 ***	−0.562	<0.001 ***
IFIS. General Fitness (Score 1–5)	3.0	(1.0)	2.0	(1.0)	2.0	(2.0)	<0.001 ***	−0.572	<0.001 ***
IFIS. Cardiorespiratory Fitness (Score 1–5)	3.0	(1.0)	3.0	(1.0)	2.0	(1.0)	0.001 *	−0.413	<0.001 ***
IFIS. Muscular Strength (Score 1–5)	3.0	(1.3)	2.0	(1.0)	2.0	(0.8)	<0.001 ***	−0.483	<0.001 ***
IFIS. Speed–Agility (Score 1–5)	3.0	(0.3)	2.0	(1.0)	2.0	(0.0)	<0.001 ***	−0.429	<0.001 ***
IFIS. Flexibility (Score 1–5)	3.0	(1.0)	2.0	(1.0)	2.5	(1.0)	0.136	−0.184	0.093
ABC Scale (Score 0–100)	69	(38)	54	(27)	46	(30)	0.029	−0.292	0.007
FES—I (Score 16–64)	25	(8)	38	(12)	49	(7)	<0.001 ***	0.570	<0.001 ***

*n* (participants); Mdn (Median); IQR (Interquartile Range); *p* (*p*-value from Mann–Whitney U test, Bonferroni correction *p* < 0.002); *p* † (*p*-value from Spearman’s rho, Bonferroni correction *p* < 0.002); * (*p* < 0.002); *** (*p* < 0.001); s (Seconds); Rep (Repetitions); m (metres); cm (centimetres); i (Imagined); Max (Maximum); IFIS (International Fitness Scale. 5: the worst self-reported fitness; 25: the best self-reported fitness. IFIS Components: Score 1–5, with 1 being the worst self-reported fitness and 5 being the best); ABC Scale (Activities-Specific Balance Confidence Scale. 0: Not confident at all; 100: completely confident); FES—I (Fall Efficacy Scale—International; 16, no concern about falling; 64, severe concern about falling).

**Table 5:** Relationships and correlations between sleep problems, physical function, perceived physical fitness, balance confidence, and fear of falling.

Variables	Sleep Problems							Correlations	
	Low (*n* = 21)		Moderate (*n* = 54)		Severe (*n* = 9)				
	Mdn	(IQR)	Mdn	(IQR)	Mdn	(IQR)	*p*	*rho*	*p* †
i-Time Up and Go test (s)	4.80	(2.09)	5.27	(2.68)	6.85	(3.25)	0.031	0.289	0.008
Time Up and Go test (s)	6.59	(1.61)	8.08	(3.45)	8.65	(5.54)	0.060	0.246	0.024
Dual Time Up and Go test (s)	7.52	(1.40)	9.63	(4.92)	10.71	(8.89)	0.014	0.316	0.003
Four Step Square test (s)	6.33	(2.52)	7.41	(2.08)	7.28	(4.06)	0.131	0.169	0.125
4 m Walking test (s)	2.47	(0.50)	2.79	(0.98)	2.70	(1.36)	0.077	0.232	0.036
30 m Walking test (s)	17.35	(3.65)	19.62	(7.19)	22.45	(10.10)	0.262	0.181	0.109
6 min Walking test (m)	483	(104)	469	(166)	467	(167)	0.592	−0.114	0.315
i-30″ Sit to Stand test (rep)	14	(9)	13	(4)	11	(6)	0.291	−0.173	0.315
30″ Sit to Stand test (rep)	11	(4)	10	(4)	9	(5)	0.176	−0.205	0.117
Dual 30″ Sit to Stand test (rep)	10	(3)	10	(4)	8	(3)	0.466	−0.130	0.062
5rep Sit to Stand test (s)	11.56	(4.00)	13.28	(4.00)	15.43	(8.00)	0.013	0.325	0.240
Max Strength Handgrip (kg)	25.60	(6.90)	21.85	(5.45)	23.30	(16.45)	0.003	−0.301	0.003
Max Strength/Weight Ratio	0.40	(0.13)	0.27	(0.09)	0.30	(0.23)	0.005	−0.338	0.005
30″ Biceps Curl test (rep)	15	(5)	13	(6)	18	(11)	0.344	−0.080	0.008
Functional reach test (cm)	36	(10)	32	(15)	36	(9)	0.463	−0.061	0.476
Chair Sit and Reach (cm)	2	(16)	−2	(14)	−6	(18)	0.038	−0.264	0.017
Back Scratch (cm)	1	(12)	−4	(14)	−9	(16)	0.159	−0.211	0.055
IFIS (Score 5–25)	14.0	(6.0)	12.0	(4.0)	11.0	(4.0)	0.019	−0.311	0.004
IFIS. General Fitness (Score 1–5)	3.0	(1.5)	2.0	(1.0)	2.0	(1.0)	0.149	−0.251	0.051
IFIS. Cardiorespiratory Fitness (Score 1–5)	3.0	(1.5)	3.0	(1.0)	3.0	(1.0)	0.135	−0.203	0.065
IFIS. Muscular Strength (Score 1–5)	3.0	(1.5)	2.0	(1.0)	2.0	(1.0)	0.020	−0.306	0.005
IFIS. Speed–Agility (Score 1–5)	3.0	(1.0)	2.0	(1.0)	2.0	(0.5)	0.099	−0.233	0.033
IFIS. Flexibility (Score 1–5)	3.0	(1.0)	2.0	(1.0)	2.0	(1.5)	0.103	−0.233	0.033
ABC Scale (Score 0–100)	69	(29)	53	(29)	46	(23)	0.019	−0.308	0.004
FES—I (Score 16–64)	28	(9)	39	(14)	44	(7)	<0.001 ***	0.471	<0.001 ***

*n* (participants); Mdn (Median); IQR (Interquartile Range); *p* (*p*-value from Mann–Whitney U test, Bonferroni correction *p* < 0.002); *p* † (*p*-value from Spearman’s rho, Bonferroni correction *p* < 0.002); *** (*p* < 0.001); s (Seconds); Rep (Repetitions); m (metres); cm (centimetres); i (Imagined); Max (Maximum); IFIS (International Fitness Scale. 5: the worst self-reported fitness; 25: the best self-reported fitness. IFIS Components: Score 1–5, with 1 being the worst self-reported fitness and 5 being the best); ABC Scale (Activities-Specific Balance Confidence Scale. 0: Not confident at all; 100: completely confident); FES—I (Fall Efficacy Scale—International. No concern about falling, 16; 64, severe concern about falling).

## Data Availability

The data can be obtained on request from the corresponding author.

## Supplementary Materials

The following supporting information can be downloaded at: https://www.mdpi.com/article/10.3390/nursrep14040207/s1, STROBE Statement—Checklist of items that should be included in reports of cross-sectional studies.

## Public Involvement Statement

No public involvement in any aspect of this research.

## Guidelines and Standards Statement

The reporting in the article adheres to the STROBE checklist for cross-sectional studies.
